# Characteristics and outcomes of out-of-hospital cardiac arrest due to drowning – a nationwide registry-based study

**DOI:** 10.1186/s13049-025-01469-1

**Published:** 2025-10-02

**Authors:** Christoph Hüser, Christine Eimer, Jan Wnent, Sadrija Cukoski, Matthias Johannes Hackl, Victor Suárez, Jan-Thorsten Gräsner, Stephan Seewald

**Affiliations:** 1 Emergency Department, University of Cologne, Faculty of Medicine and University Hospital Cologne, Cologne, Germany; 2 Department II of Internal Medicine and Center for Molecular Medicine Cologne, University of Cologne, Faculty of Medicine and University Hospital Cologne, Cologne, Germany; 3 Department of Anaesthesiology and Intensive Care Medicine, University Hospital Schleswig-Holstein, Campus Kiel, Kiel, Germany; 4 Institute for Emergency Medicine, University Hospital Schleswig-Holstein, Kiel, Germany

**Keywords:** German Resuscitation Registry, Emergency medicine, Emergency medical services, EMS, Hypothermia, Drowning

## Abstract

**Aim of the study:**

To describe and compare cases of resuscitation after out-of-hospital cardiac arrest (OHCA) attributed to drowning (D-OHCA) versus other causes (ND-OHCA).

**Methods:**

Retrospective, descriptive and comparative analysis of D-OHCA vs. ND-OHCA patients registered in the German Resuscitation Registry from January 2013 to December 2023 using Chi-square, Mann-Whitney U tests and regression analysis. Key variables included 10-year age groups, body temperature measured at the scene, prehospital factors (e.g., bystander CPR, initial rhythm), and outcomes such as survival and neurological status (CPC, mRS) at hospital discharge.

**Results:**

Of the 68,719 included patients 316 (0.5%) had D-OHCA with 50% of the cases occurring during the summer months (June, July and August). D-OHCA in comparison to ND-OHCA patients were younger (median age 50 years vs. 72.5 years, *p* < 0.001), had a higher rate of asystole as initial rhythm (73.1 vs. 54.9%, *p* < 0.001) and a lower initial body temperature (median of 31.1 °C vs. 35.8 °C, *p* < 0.001). While overall survival and favourable neurological outcomes did not differ significantly between groups, stratified analysis showed that D-OHCA patients aged 0–10 years had significantly higher survival rates (44.7% vs. 16.3%, *p* < 0.001) and favourable neurological outcomes at hospital discharge (34.0% vs. 7.6%, *p* < 0.001) compared to ND-OHCA patients under 11 years.

**Conclusion:**

Drowning was a rare cause of out-of-hospital cardiac arrest in this study, often occurring during summer months. Outcome in D-OHCA was generally comparable to ND-OHCA and only better in children aged up to 10 years. Lower body temperatures were associated with unfavourable outcomes in most D-OHCA cases.

**Supplementary Information:**

The online version contains supplementary material available at 10.1186/s13049-025-01469-1.

## Background

According to the WHO, drowning is one of the 5 leading causes of death among 1–14 year olds in Europe [1]. Most drowning deaths are preventable through measures such as awareness campaigns or water competency training [1, 2] and resuscitation is only necessary in a minority of drowning incidents [3]. Out-of-hospital cardiac arrest due to drowning (D-OHCA) in children has been reported to have more favourable outcomes in comparison to adults [4, 5], possibly based on different characteristics such as lower rates of comorbidities, higher rates of witnessed arrest and bystander CPR [5].

Meanwhile, factors influencing outcome in D-OHCA have been found to be heterogeneous and partly contradictory in different reviews [6–8], so that knowledge and evidence on resuscitation in drowning is limited [9, 10]. This is especially important, as D-OHCA patients are often hypothermic, so that prolonged and resource-intensive cardiopulmonary resuscitation (CPR) efforts until rewarming are recommended [10, 11]. But if these result in a favourable outcome remains questionable [12].

Therefore, the aim of this study was to create a better understanding and evidence for resuscitation due to drowning in Germany by comparing epidemiological parameters and prognostic factors for patients registered in the German Resuscitation Registry (GRR), which received CPR due to drowning (D-OHCA) versus other causes (ND-OHCA) with a focus on age and initial body temperature. Additionally, we wanted to evaluate neurological outcomes in patients with prolonged cardiopulmonary resuscitation > 20 min and primary asystole.

## Methods

### Study design

We performed a retrospective, descriptive and comparative analysis of patients with OHCA registered anonymously in the German Resuscitation Registry (GRR) [13] from January 2013 to December 2023. This was approved by the ethics commission of the Christian-Albrechts-University Kiel (Ref. no.: D 573/22) and the scientific advisory board of the GRR (Ref. no.: 2022-05). Informed consent was not required.

### German emergency medical services (EMS)

EMS in Germany is based on a two-tier system consisting of one unit with paramedics (e.g. an ambulance) and a second unit that is physician staffed. They are simultaneously dispatched for a CPR call. Staff from both units are trained in emergency medicine procedures and providing advanced cardiac life support in accordance with current guidelines. The physicians are allowed to terminate the resuscitation on scene.

### German resuscitation registry

The GRR comprises voluntary and anonymously submitted data from 146 German EMS serving approximately 39 million of the 82 million people living in Germany [14]. To ensure high data quality the database is implemented with multiple plausibility checks and includes a ‘prehospital dataset’ as well as a ‘post resuscitation care’ dataset.

The ’prehospital dataset’ comprises 118 variables focussing on prehospital issues, such as presumed aetiology, primary rhythm or use of IO-access, based on the EMS physician´s protocol (Table S1). It also includes the body temperature measured by EMS staff on scene as initial body temperature. After documentation in the GRR, the data are reviewed by the physician responsible for the EMS, followed by approval in the registry. The general health condition of the patients before the cardiac arrest is assessed using the pre emergency score (PES). The score describes the state of health analogous to the American Society of Anaesthesiologists categories (1: without previous disease, 2: with mild systemic disease, 3: with severe systemic disease, 4: normal daily life impossible; severe systemic disease that is a constant threat to life, 5: moribund patient who is not expected to survive the next 24 h) [15]. The cause of OHCA is determined by the EMS team´s judgement.

The ‘post resuscitation care’ dataset contains in-hospital post-resuscitation efforts and patients’ in-hospital outcome with the participating hospitals being able to choose between a basic and a detailed version with 156 variables [16]. It includes the Cerebral Performance Categories (CPC) (1: good cerebral performance; 2: moderate cerebral disability; 3: severe cerebral disability 4: coma or vegetative state; 5: brain death) or the modified Rankin-scale (mRS) (0: no symptoms; 1: no significant disability; 2: slight disability; 3: moderate disability; 4: moderately severe disability; 5: severe disability; 6: dead) to define the neurological outcome of the patient [17]. The CPC or mRS was assigned by the treating physician at the time of hospital discharge.

Not all EMS report in-hospital data due to local data regulations. With the intention to still present valid results the GRR steering committee selects a group of EMS which report outcome data as “good data quality group” defined by: an incidence above 30/100,000 inhabitants per year, ROSC in less than 80% of cases, information about the ROSC-after-cardiac-arrest score [18] in more than 60% of the cases, and, if relevant, documented hospital care available for more than 30% of the cases. These criteria were established in 2017 by decision of the steering committee and have since been applied uniformly across all research projects.

### Inclusion and exclusion criteria

We included patients from the GRR with good data quality, who received prehospital resuscitation due to drowning or all other reasons (including cardiac cause, hypoxia, trauma, exsanguination, intracranial bleeding, stroke, metabolic cause, sepsis and poisoning). The cause of OHCA was defined as reported to the GRR by the EMS team´s initial judgement. Patients who received resuscitation but were declared dead at the scene and not transported to the hospital were included in the analysis.

### Outcome

The primary outcome parameters were survival at hospital discharge and a favourable neurological outcome, defined as a CPC of 1 or 2 or mRS of 0,1 or 2 at hospital discharge.

### Statistics

For the descriptive analysis of numerical variables, the median and interquartile range (IQR), for categorial and dichotomous variables the frequency and proportion in percent were calculated.

For comparative analysis Mann-Whitney-U or Chi-square tests (respectively Fisher´s exact test for small sample sizes) were used if applicable. Tests were two-sided. A *p*-value of 0.05 or less was considered statistically significant. To analyse the risk adjusted influence of drowning on survival with favourable neurological function, a logistic regression analysis, with favourable neurological outcome as dependent variable and potential predictors associated with outcome [18] as independent variables, were performed. If the percentage of missing values in one variable was low and the survival rate was near to the average, we included that cases in the reference category (location of cardiac arrest and pre emergency status). Otherwise missing data were included as a separate category of each variable in the multivariate regression (time between collapse and start of CPR). This category was assigned the reference category, so that the coefficient for this category was automatically set to zero. The amount of adrenaline administered served as a surrogate parameter for the duration of resuscitation.

Analysis was conducted across all age groups, with additional stratification into 10-year age intervals to account for potential age-related differences in patient characteristics and outcomes, particularly in children up to 10 years.

In addition, prognostic factors for survival at hospital discharge with favourable neurological function of drowning and non-drowning patients were investigated using univariate analyses. Calculations were performed with IBM SPSS Statistics 29 (Armonk, NY, USA).

## Results

**Fig. 1:**
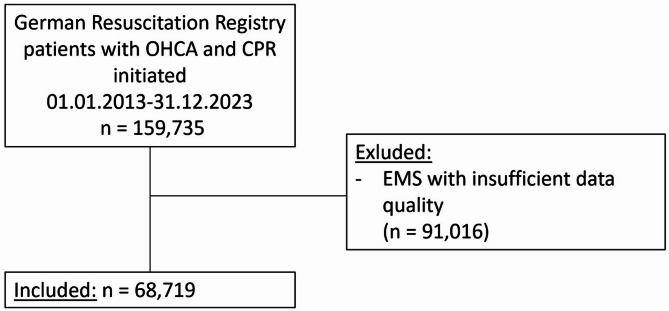
Strobe inclusion flow chart

A total of 68,719 patients were included in the analysis (Fig. 1), of which 316 (0.5%) had OHCA attributed to drowning. Demographic and baseline characteristics are shown in Table 1. Patients with D-OHCA were younger (median age 50 years vs. 72.5 years, *p* < 0.001) and had a lower PES, indicating less preexisting diseases compared with ND-OHCA patients. Patients aged 0–10 years made up 14.9% of the patients with D-OHCA, while this age group constituted only 1.0% of patients with ND-OHCA. D-OHCA was significantly less often witnessed by bystanders (27.2% vs. 42.7%, *p* < 0.001), but bystander CPR was more common in D-OHCA (53.8% vs. 39.3%, *p* < 0.001). Nonetheless, the time between collapse and start of CPR was longer with D-OHCA (median 10 min vs. 7 min, *p* < 0.001). Drowning patients had a lower rate of shockable initial rhythm (7.9% vs. 22.1%, *p* < 0.001) and higher proportion of asystole as initial rhythm (73.1 vs. 54.9%, *p* < 0.001). They also had a lower initial body temperature (median of 31.1 °C vs. 35.8 °C, *p* < 0.001).

**Table 1:** Demographics and patients´ characteristics

	D-OHCA patients	ND-OHCA patients	*p*
	*n* = 316	*n* = 68,403	
*age*	*median (IQR)*	*median (IQR)*	
years	50 (23.9;72.4)	72.5 (60.2;82.0)	*p* < 0.001
	*n (%)*	*n (%)*	
0–10 years	47 (14.9)	683 (1.0)	
11–20 years	17 (5.4)	446 (0.7)	
21–30 years	33 (10.4)	999 (1.5)	
31–40 years	26 (8.2)	1832 (2.7)	
41–50 years	32 (10.1)	4223 (6.2)	
51–60 years	31 (9.8)	9733 (14.2)	
61–70 years	38 (12.0)	13,755 (20.1)	
71–80 years	41 (13.0)	17,166 (25.1)	
81 + years	41 (13.0)	19,050 (27.8)	*p* < 0.001
missing	10 (3.2)	516 (0.7)	
*sex*			
male	222 (70.3)	44,811 (65.5)	
female	94 (29.7)	23,575 (34.5)	*p* = 0.078
*pre emergency status (PES)*			
PES 1	118 (37.3)	5,627 (8.2)	
PES 2	50 (15.8)	16,468 (24.1)	
PES 3	44 (13.9)	26,447 (38.7)	
PES 4	3 (0.9)	6,224 (9.1)	
PES 5	0 (0.0)	248 (0.4)	*p* < 0.001
unknown	101 (32.0)	13,389 (19.6)	
*presumed etiology of CA*			
Drowning	316 (100.0)	0 (0.0)	
Hypoxia	0 (0.0)	9,439 (13.8)	
Cardiac	0 (0.0)	41,235 (60.3)	
Other causes	0 (0.0)	8,545 (12.5)	*p* < 0.001
unknown	0 (0.0)	9,184 (13.4)	
*location of arrest*			
home	53 (16.8)	43,858 (64.1)	
nursing home	1 (0.3)	7,047 (10.3)	
working place	0 (0.0)	1,351 (2.0)	
doctor´s office	0 (0.0)	1,072 (1.6)	
public place	154 (48.7)	4,278 (6.3)	
medical institution	0 (0.0)	1,308 (1.9)	
sports facility	36 (11.4)	627 (0.9)	
mass event	1 (0.3)	75 (0.1)	
others	70 (22.1)	8,371 (12.2)	
unknown	1 (0.3)	416 (0.6)	*p* < 0.001
*witnessed*			
no	228 (72.2)	29,370 (42.9)	
lay person	86 (27.2)	29,194 (42.7)	
EMS	2 (0.6)	9,839 (14.4)	*p* < 0.001
*bystander CPR*			
yes	170 (53.8)	26,915 (39.3)	*p* < 0.001
*Time between collapse and start of CPR (min)*			
median (IQR)	10.0 (5.0;16.5)	7.0 (3.0;10.0)	
missing	*n* = 127	*n* = 19,246	*p* < 0.001
*First documented rhythm (on scene)*			
VF/pVT	25 (7.9)	15,113 (22.1)	
PEA	40 (12.7)	15,245 (22.3)	
asystole	231 (73.1)	37,542 (54.9)	
unknown	20 (6.3)	503 (0.7)	*p* < 0.001
*Initial temperature (°C)*			
median (IQR)	31.1 (27.1;34.4)	35.8 (35.0;36.4)	*p* < 0.001
< 24	19 (6.0)	56 (0.1)	
24 - <28	21 (6.6)	126 (0.2)	
28 - <32	29 (12.3)	590 (0.9)	
32–35	37 (11.7)	3,851 (5.6)	
> 35	30 (9.5)	12,267 (17.9)	
missing	170 (53.8)	51,513 (75.3)	*p* < 0.001
*duration of CPR efforts until death (min)*			
median (IQR)	28.0 (15.5;42.0)	28.0 (19.0;39.0)	*p* = 0.787
missing	*n* = 81	*n* = 40,497	
*duration of CPR until first ROSC (min)*			
median (IQR)	18.5 (9;28.3)	18.0 (10.0;26.0)	*p* = 0.750
missing	*n* = 94	*n* = 47,532	

CA: cardiac arrest, CPR: cardiopulmonary resuscitation, EMS: Emergency Medical Services, IQR: interquartile range, min: minutes, D-OHCA: out-of-hospital cardiac arrest attributed to drowning, ND-OHCA: out-of-hospital cardiac arrest not attributed to drowning, PEA: pulseless electrical activity, pVT: pulseless ventricular tachycardia, ROSC: return of spontaneous circulation, VF: ventricular fibrillation

Outcome parameters are presented in Table 2. D-OHCA patients were transported and admitted to the hospital more often with ongoing CPR (29.7% vs. 11.2%, *p* < 0.001) and more often had ROSC at hospital admission (39.6% vs. 35.0%, *p* < 0.001). For patients with ongoing CPR at hospital admission, survival rates (3.2% vs. 4.0%, *p* = 0.684) and favourable neurological outcome rates (2.1% vs. 2.4%, *p* = 0.879) were comparable in D-OHCA (*n* = 94) and ND-OHCA (*n* = 7,688, results not shown). In D-OHCA patients with CPR > 20 min and primary asystole 2.6% (2 of 78) had a favourable neurological outcome, versus 0.6% (110 of 18,363) after non-drowning OHCA (*p* = 0.026, results not shown).

**Table 2:** Patients’ outcome

	Hospital admission			Survival	Favourable Outcome	
	with ROSC	with ongoing CPR		at hospital discharge	at hospital discharge#	
	n (%)	n (%)		n (%)	n (%)	
***all ages***						
D-OHCA	125 (39.6)	94 (29.7)		47 (14.9)	32 (10.1)	
ND-OHCA	23,948 (35.0)	7,688 (11.2)		8,057 (11.8)	5,670 (8.3)	
	*p* < 0.001			*p* = 0.093	*P* = 0.259	
***age groups***						
*0–10 years*						
D-OHCA (*n* = 47)	32 (68.1)	14 (29.8)		21 (44.7)	16 (34.0)	
ND-OHCA (*n* = 683)	253 (37.0)	150 (22.0)		111 (16.3)	52 (7.6)	
	*p* < 0.001			*p* < 0.001	*p* < 0.001	
*11–20 years*						
D-OHCA (*n* = 17)	9 (52.9)	5 (29.4)		4 (23.5)	2 (11.8)	
ND-OHCA (*n* = 446)	166 (37.2)	122 (25.1)		77 (17.3)	40 (9.0)	
	*p* = 0.227			*p* = 0.515	*p* = 0.660	
*21–30 years*						
D-OHCA (*n* = 33)	11 (33.3)	11 (33.3)		2 (6.1)	1 (3.0)	
ND-OHCA (*n* = 999)	390 (39.0)	190 (19.0)		208 (20.8)	145 (14.5)	
	*p* = 0.123			*p* = 0.045	*p* = 0.073	
*31–40 years*						
D-OHCA (*n* = 26)	10 (38.5)	8 (30.8)		2 (7.7)	0 (0.0)	
ND-OHCA (*n* = 1,832)	704 (38.4)	352 (19.2)		360 (19.7)	247 (13.5)	
	*p* = 0.275			*p* = 0.208	*p* = 0.040	
*41–50 years*						
D-OHCA (*n* = 32)	8 (25.0)	13 (40.6)		4 (12.5)	3 (9.4)	
ND-OHCA (*n* = 4,223)	1,662 (39.4)	822 (19.5)		899 (21.3)	643 (15.2)	
	*p* = 0.010			*p* = 0.282	*p* = 0.464	
*51–60 years*						
D-OHCA (*n* = 31)	11 (35.5)	13 (41.9)		3 (9.7)	3 (9.7)	
ND-OHCA (*n* = 9,733)	3,919 (40.3)	1,581 (16.2)		1,845 (19.0)	1,374 (14.1)	
	*p* < 0.001			*p* = 0.252	*p* = 0.612	
*61–70 years*						
D-OHCA (*n* = 38)	12 (31.6)	11 (28.9)		4 (10.5)	3 (7.9)	
ND-OHCA (*n* = 13,755)	5,387 (39.2)	1,754 (12.8)		2,027 (14.7)	1,420 (10.3)	
	*p* = 0.012			*p* = 0.646	*p* = 0.793	
*71–80 years*						
D-OHCA (*n* = 41)	16 (39.0)	9 (22.0)		4 (9.8)	2 (4.9)	
ND-OHCA (*n* = 17,166)	6,072 (35.4)	1,635 (9.5)		1,630 (9.5)	1,115 (6.5)	
	*p* = 0.013			*p* = 0.794	*p* = 0.675	
*81–90 years*						
D-OHCA (*n* = 32)	10 (31.3)	5 (15.6)		2 (6.3)	1 (3.1)	
ND-OHCA (*n* = 15,774)	4,579 (29.0)	923 (5.9)		802 (5.1)	559 (3.5)	
	*p* = 0.051			*p* = 0.677	*p* = 0.898	
*91 + years*						
D-OHCA (*n* = 9)	4 (44.4)	3 (33.3)		0 (0.0)	0 (0.0)	
ND-OHCA (*n* = 3,276)	689 (21.0)	102 (3.1)		79 (2.4)	57 (1.7)	
	*p* < 0.001			*p* = 0.637	*p* = 0.690	

#Included hospital discharged with CPC 1 and 2 or mRS 0, 1 or 2

CPC: cerebral performance categories, CPR: cardiopulmonary resuscitation, D-OHCA: out-of-hospital cardiac arrest attributed to drowning, mRS: modified rankin score, ND-OHCA: out-of-hospital cardiac arrest not attributed to drowning, ROSC: Return of spontaneous circulation

While an overall comparison between D-OHCA and ND-OHCA patients showed a non-statistically significant trend toward survival and favourable neurological outcomes in the drowning group, age-stratified analysis revealed that this trend was solely driven by children aged 0–10 years. This group had significantly higher chances of survival (44.7% vs. 16.3%, *p* < 0.001) and favourable neurological outcome at hospital discharge (34.0% vs. 7.6% *p* < 0.001) compared to ND-OHCA patients up to 10 years. In contrast, for all other age groups from 21 to 70 years, D-OHCA patients had lower survival and neurological outcome rates, though these differences were mostly not statistically significant (Table 2).

D-OHCA patients aged up to 10 years had a lower PES (80.9% PES 1 vs. 29.3% PES 1, *p* < 0.001), higher bystander CPR rates (91.5% vs. 47.9%, *p* < 0.001) and more often bradycardia as initial rhythm (25.5% vs. 0.4%, *p* < 0.001) in comparison to D-OHCA patients aged ≥ 11 years (Table 3).

**Table 3:** D-OHCA characteristics according to age

D-OHCA	≤ 10 years	11 years and older	*p*
	*n* = 47	*n* = 259	
*sex*			
male	23 (48.9)	194 (74.9)	
female	24 (51.1)	65 (25.1)	*p* < 0.001
*pre emergency status (PES)*			
PES 1	38 (80.9)	76 (29.3)	*p* < 0.001
PES 2	0 (0)	50 (19.3)	
PES 3	1 (2.1)	43 (16.6)	
PES 4	0 (0)	3 (1.2)	
unknown	8 (17.0)	87 (33.6)	
*location of arrest*			
home	11 (23.4)	42 (16.2)	0.660
nursing home	0 (0)	1 (0.4)	
public place	20 (42.6)	144 (55.6)	
sports facility	8 (17.0)	28 (10.8)	
mass event	0 (0)	1 (0.4)	
others	8 (17.0)	43 (16.6)	
*witnessed*			
no	36 (76.6)	183 (70.7)	
lay person	11 (23.4)	74 (28.6)	
EMS	0 (0)	2 (0.8)	0.625
*bystander CPR*			
yes	43 (91.5)	124 (47.9)	*p* < 0.001
*Time between collapse and start of CPR (min)*			
median (IQR)	5.0 (2.0;12.0)	10.0 (5.5;10.0)	
missing	*n* = 35	*n* = 90	0.134
*First documented rhythm (on scene)*			
VF/pVT	1 (2.1)	24 (9.3)	
PEA	6 (12.8)	34 (13.1)	
asystole	28 (59.6)	196 (75.7)	
bradycardia	12 (25.5)	1 (0.4)	
unknown	0 (0)	4 (1.6)	*p* < 0.001
*Initial temperature (°C)*			
median (IQR)	33.0 (29.9;35.8)	30.6 (27.0;34.5)	
missing	*n* = 25	*n* = 138	0.100
< 24	2 (4.3)	17 (6.6)	
24 - <28	2 (4.3)	19 (7.3)	
28 - <32	4 (8.5)	33 (12.7)	
32–35	8 (17.0)	28 (10.8)	
> 35	6 (12.8)	24 (9.3)	
missing	25 (53.2)	138 (53.3)	0.657
*duration of CPR efforts until death (min)*			
median (IQR)	27.0	29.0 (18.0;43.0)	*p* = 0.873
missing	*n* = 46	*n* = 184	
*duration of CPR until first ROSC (min)*			
median (IQR)	10.0 (4.0;39.0)	19.5 (11.3;27.0)	*p* = 0.132
missing	*n* = 22	*n* = 192	

CA: cardiac arrest, CPR: cardiopulmonary resuscitation, D-OHCA: out-of-hospital cardiac arrest attributed to drowning, EMS: Emergency Medical Services, IQR: interquartile range, min: minutes, PEA: pulseless electrical activity, pVT: pulseless ventricular tachycardia, ROSC: return of spontaneous circulation, VF: ventricular fibrillation

In the univariate analyses performed, the prognostic factors influencing a favourable neurological outcome at hospital discharge were comparable in D-OHCA and ND-OHCA (Table 4a and 4b). In comparison with ND-OHCA, there was a higher proportion of patients up to 10 years of age (51.6% vs. 0.9%) and a higher proportion of non-shockable cardiac arrhythmias (75% vs. 31.5%) in D-OHCA with a favourable neurological outcome. A lower body temperature was associated with unfavourable outcomes in D-OHCA.

**Table 4a:** Univariate analysis of prognostic factors for favourable neurological function (CPC 1 / 2 or mRS 0, 1 and 2) at hospital discharge after D-OHCA

D-OHCA	Favourable Outcome#	Unfavourable Outcome*	*p*-value
	*n* = 32	*n* = 284	
	*n* (%)	*n* (%)	
*sex*			
male	15 (46.9)	207 (72.9)	**0.004**
female	17 (53.1)	77 (27.1)	
*pre emergency status*			
unknown	6 (18.8)	95 (33.5)	**0.015**
PES 1	21 (65.6)	97 (34.2)	
PES 2	3 (9.4)	47 (16.5)	
PES 3	2 (6.3)	42 (14.8)	
PES 4	0	3 (1.1)	
PES 5	0	0	
*age (missing n = 10)*			
0–10 years	16 (51.6)	31 (11.3)	
11–20 years	2 (6.5)	15 (5.5)	
21–30 years	1 (3.2)	32 (11.6)	
31–40 years	0	26 (9.5)	
41–50 years	3 (9.7)	29 (10.5)	
51–60 years	3 (9.7)	28 (10.2)	**< 0.001**
61–70 years	3 (9.7)	35 (12.7)	
71–80 years	2 (6.5)	39 (14.2)	
81–90 years	1 (3.2)	31 (11.3)	
91 + years	0	9 (3.3)	
*age (years) (missing n = 10)*			
median (IQR)	10.4 (2.6;53.5)	53.5 (28.1;74.3)	**< 0.001**
*location of arrest*			
home	2 (6.3)	51 (18.0)	
nursing home	0	1 (0.4)	
working place	0	0	
doctor´s office	0	0	
public place	13 (40.6)	141 (49.6)	**< 0.001**
medical institution	0	0	
sports facility	13 (40.6)	23 (8.1)	
mass event	0	1 (0.4)	
others	4 (12.5)	66 (23.2)	
unknown	0	1 (0.4)	
*witnessed*			
no	17 (53.1)	211 (74.3)	**0.029**
lay person	15 (46.9)	71 (25.0)	
EMS	0	2 (0.7)	
*First documented rhythm (on scene) (missing n = 20)*			
VF/pVT	5 (25.0)	20 (7.2)	**< 0.001**
PEA	6 (30.0)	34 (12.3)	
asystole	9 (45.0)	222 (80.4)	
*bystander CPR*			
yes	25 (78.1)	145 (51.1)	**0.004**
no	7 (21.9)	139 (48.9)	
*Administration of adrenaline*			
unknown	1 (3.1)	1 (0.4)	**< 0.001**
no	23 (71.9)	59 (20.8)	
<2 mg	3 (9.4)	45 (15.8)	
2-<4 mg	1 (3.1)	61 (21.5)	
4-<6 mg	1 (3.1)	49 (17.3)	
6-<8 mg	1 (3.1)	27 (9.5)	
>=8 mg	2 (6.3)	42 (14.8)	
*Initial temperature (°C) (missing n = 170)*			
median (IQR)	34.9 (33.5;36.4)	30.6 (26.9;34.1)	**< 0.001**
*Initial temperature (°C)*			
<24	0	19 (6.7)	**0.019**
24 - <28	1 (3.1)	20 (7.0)	
28 - <32	2 (6.3)	37 (13.0)	
32–35	5 (15.6)	32 (11.3)	
>35	8 (25.0)	22 (7.7)	
missing	16 (50.0)	154 (54.2)	
*Time between collapse and start of CPR (min) (missing n = 127)*			
median (IQR)	7 (2;12.5)	10 (6;17)	**0.040**

#Included hospital discharged with CPC 1 and 2 or mRS 0, 1 or 2

*Included died on scene, died within 24 h, died in hospital, hospital discharged with unfavourable neurological outcome (CPC 3–4 or mRS 3–5), missing outcome information

CPC: cerebral performance categories, CPR: cardiopulmonary resuscitation, D-OHCA: out-of-hospital cardiac arrest attributed to drowning, EMS: Emergency Medical Services, mRS: modified rankin score, PEA: pulseless electrical activity, pVT: pulseless ventricular tachycardia, VF: ventricular fibrillation

**Table 4b:** Univariate analysis of prognostic factors for favourable neurological function (CPC 1 / 2 or mRS 0, 1 and 2) at hospital discharge after ND-OHCA

ND-OHCA	Favourable Outcome#	Unfavourable Outcome *	*p*-value
	*n* = 5,670	*n* = 62,733	
	*n* (%)	*n* (%)	
*Sex (missing n = 17)*			
male	4,189 (73.9)	40,622 (64.8)	**<0.001**
female	1,481 (26.1)	22,094 (35.2)	
*pre emergency status*			
unknown	1,036 (18.3)	12,353 (19.7)	**<0.001**
PES 1	1,007 (17.8)	4,620 (7.4)	
PES 2	2,145 (37.8)	14,323 (22.8)	
PES 3	1,349 (23.8)	25,098 (40.0)	
PES 4	130 (2.3)	6,094 (9.7)	
PES 5	3 (0.1)	245 (0.4)	
*age (missing n = 516)*			
0–10 years	52 (0.9)	631 (1.0)	
11–20 years	40 (0.7)	406 (0.7)	
21–30 years	145 (2.6)	854 (1.4)	
31–40 years	247 (4.4)	1,585 (2.5)	
41–50 years	643 (11.4)	3,580 (5.8)	
51–60 years	1,374 (24.3)	8,359 (13.4)	**< 0.001**
61–70 years	1,420 (25.1)	12,335 (19.8)	
71–80 years	1,115 (19.7)	16,051 (25.8)	
81–90 years	559 (9.9)	15,215 (24.4)	
91 + years	57 (1.0)	3219 (5.2)	
*age (years) (missing n = 18)*			
median (IQR)	63.3 (53.5;73.8)	73.4 (61.1;82.5)	**< 0.001**
*location of arrest*			
home	2,831 (49.9)	41,027 (65.4)	
nursing home	175 (3.1)	6,872 (11.0)	
working place	297 (5.2)	1,054 (1.7)	
doctor´s office	234 (4.1)	838 (1.3)	
public place	704 (12.4)	3,574 (5.7)	**< 0.001**
medical institution	111 (2.0)	1,197 (1.9)	
sports facility	234 (4.1)	393 (0.6)	
mass event	20 (0.4)	55 (0.1)	
others	1040 (18.3)	7,331 (11.7)	
unknown	24 (0.4)	392 (0.6)	
*witnessed*			
no	895 (15.8)	28,475 (45.4)	**< 0.001**
lay person	3,410 (60.1)	25,784 (41.1)	
EMS	1,365 (24.1)	8,474 (13.5)	
*First documented rhythm (on scene) (missing n = 503)*			
VF/pVT	3,806 (68.4)	11,307 (18.1)	**< 0.001**
PEA	1,037 (18.6)	14,208 (22.8)	
asystole	719 (12.9)	36,823 (59.1)	
*bystander CPR*			
yes	2,750 (48.5)	24,165 (38.5)	**< 0.001**
no	2,920 (51.5%)	38,568 (61.5)	
*Administration of adrenaline*			
unknown	40 (0.7)	437 (0.7)	**< 0.001**
no	3,014 (53.2)	14,059 (22.4)	
<2 mg	1,065 (18.8)	7,369 (11.7)	
2-<4 mg	852 (15.0)	14,409 (23.0)	
4-<6 mg	364 (6.4)	11,813 (18.8)	
6-<8 mg	174 (3.1)	6,229 (9.9)	
>=8 mg	161 (2.8)	8.417 (13.4)	
*Initial temperature (°C) (missing n = 51*,*513)*			
median (IQR)	36.0 (35.5;36.5)	35.8 (34.9;36.4)	**< 0.001**
*Initial temperature (°C)*			
<24	3 (0.1)	53 (0.1)	**< 0.001**
24 - <28	5 (0.1)	121 (0.2)	
28 - <32	10 (0.2)	580 (0.9)	
32–35	240 (4.2)	3,611 (5.8)	
>35	1,444 (25.5)	10,823 (17.3)	
missing	3,968 (70.0)	47,545 (75.8)	
*Time between collapse and start of CPR (min) (missing n = 19*,*246)*			
median (IQR)	2 (1;7)	7 (3;10)	**< 0.001**

#Included hospital discharged with CPC 1 and 2 or mRS 0, 1 or 2

*Included died on scene, died within 24 h, died in hospital, hospital discharged with unfavourable neurological outcome (CPC 3–4 or mRS 3–5), missing outcome information

CPC: cerebral performance categories, CPR: cardiopulmonary resuscitation, EMS: Emergency Medical Services, mRS: modified rankin score, ND-OHCA: out-of-hospital cardiac arrest not attributed to drowning, PEA: pulseless electrical activity, pVT: pulseless ventricular tachycardia, VF: ventricular fibrillation

The proportion of drowning as cause of OHCA remained stable over the years and 50.0% of cardiac arrest cases due to drowning happened during the summer months of June, July and August (Table S2). D-OHCA patients in the summer had a higher initial body temperature and a non-significant trend to more favourable neurological outcomes (Table S3).

In the multivariate regression analysis, D-OHCA was not found to be a significant predictor of hospital discharge with favourable neurological outcome (Table S4).

## Discussion

In this retrospective registry-based study, drowning was a rare cause of OHCA in Germany. The proportion of drowning OHCA was comparable with the proportions found in other recent European studies [19–21]. Compared to ND-OHCA cases, drowning patients were younger and had lower body temperatures measured at the scene. However, increased chances of survival and favourable neurological outcomes in D-OHCA were observed only in the 0–10 year age group. A lower body temperature at the scene was associated with decreased survival and less favourable neurological outcomes in D-OHCA patients.

Among children up to 10 years of age, 14.9% (or 1 in 15) of OHCA cases were attributed to drowning. Conversely, this age group accounted for 6.4% of all D-OHCA cases in the overall cohort. Between 11 and 30 years of age, 1 in 30 cardiac arrests was due to drowning and with advancing age drowning became a much rarer cause of arrest. In comparison, in one recent study from Denmark, children and young persons from 0 to 24 years accounted for 16% of drowning arrests [20]. This finding emphasizes that children aged 0–10 years were disproportionately more frequently affected by cardiac arrest due to drowning in our study, underscoring the urgent need to optimise prevention strategies targeting this age group in Germany. With the majority of cases happening during the summer months, as found in other studies [20–22], focus should not only be on life guards in public pools or natural swimming areas, but enhancing water competency training in young children [2] and home pool safety [23].

In general, patients with D-OHCA were not found to have a higher chance of survival or favourable neurological outcome at hospital discharge in this study. For the age decades between 21 and 70 years there was even trend that D-OHCA patients had lower chances of survival and favourable neurological outcome at hospital discharge than ND-OHCA patients. This is in contrast to other international studies, which showed comparable [22] or higher chances of survival in D-OHCA patients [19, 20]. In particular one Danish study found that both 30-day and 1-year survival were significantly higher compared to ND-OHCA (33% vs. 14% and 32% vs. 13%, respectively, *p* < 0.001) [20]. With comparable rates of preclinical death and ROSC at hospital admission between the studies as well as comparable influencing factors in the Danish study, this suggests that other, not reported factors such as submersion time must be different in the studies. In comparison, a French study found a ROSC rate for 40.6% at hospital admission, but a survival rate of only 9.0% at hospital discharge, which is more comparable to our findings [24].

Despite this, D-OHCA children aged 0–10 years showed higher chances of survival and favourable neurological outcome at hospital discharge than ND-OHCA children in this study. D-OHCA patients up to 10 years in comparison to older D-OHCA patients had higher rates of bystander CPR, a lower PES indicating better health status before the drowning event and a higher rate of bradycardia as initial rhythm. Both the high rate of bystander CPR as well as bradycardia as initial rhythm could indicate that children up to 10 years were rescued more quickly and had shorter submersion times. The outcome after OHCA due to drowning for children has similarly been better in other studies [5, 20], which was also attributed to more immediate rescue [25, 26], higher rates of bystander CPR as well as protective physiologic mechanisms in this age group [5].

An interesting finding was, that surviving D-OHCA patients showed a higher body temperature than non-survivors and only 1 out of 40 drowning patients with a body temperature < 28 °C survived with favourable neurological outcome. This might be a counterintuitive finding as case reports and a recent prominently published narrative review might lead to the impression that hypothermia can be neuroprotective and lead to a more favourable outcome after drowning [11]. However, the literature is conflicting and systematic data seem to show quite the opposite. A meta-analysis found no protective effect of low water temperature [6] and the association of hypothermia with an unfavourable outcome in drowning resuscitation has been shown before [27]. Hence, recommendations for prolonged resuscitation have been questioned [28]. For hypothermia to improve outcome, it seems crucial that protective cooling takes place before critical brain hypoxia, but drowning is often due to asphyxia before relevant hypothermia develops [11, 29]. Although favourable neurological outcomes have been reported in selected case reports or smaller case series of hypothermic drowning patients [30–36] - particularly in children - these are likely exceptional. A larger case series from the Netherlands reporting results of 98 hypothermic drowned children with CPR ≥ 30 min reported a survival rate of 11%, but all survivors had a CPC of ≥ 4 [12]. Similarly, a recent Danish study also demonstrated poor outcomes associated with hypothermia in drowning associated cardiac arrest [37].

Taken together, the likelihood of a favourable neurological outcome after prolonged resuscitation even in hypothermic D-OHCA patients appears to be limited to rare cases - particularly when protective cooling precedes cerebral hypoxia, as may occur during drowning in extremely cold water (≤ 6 °C) [11, 29]. In the majority of cases however, a lower body temperature might be more reflective of a prolonged submersion time – an indicator of an unfavourable outcome - rather than indicative for a neuroprotective effect of hypothermia occurring before hypoxia. Even during European winter, the cooling effect of water to unfold neuroprotective effects might be limited, as outcome for drowning victims from December to February was worse in a Danish study [20], children with CPR ≥ 30 min had an unfavourable neurologic outcome independent of the season [12] and in our study D-OHCA patients outside the summer months had lower initial body temperatures, but also a non-significant trend for worse neurological outcomes.

Yet, the critical question on how to identify the cases of hypothermic drowning patients that have had neuroprotective cooling and benefit from extended resuscitation during rewarming remains unsolved, although scores such as the HOPE score [38] might help to predict outcome. Of note, in all patients receiving CPR > 20 min with initial asystole, we found a significant higher rate of a favourable neurological outcome in the drowning group (2.6% vs. 0.6%; *p* = 0.026), underlining that prolonged resuscitation efforts might be justified in certain drowning patients. Further research is needed to help guide decision making on which D-OHCA patients profit from extended resuscitation efforts.

### Limitations

One limitation of this study is the retrospective and registry based design. Even though only cases with good data quality were included, a relevant proportion of data points were missing for some variables, especially for initial body temperature (missing in 53.8% of D-OHCA and 75.3% of ND-OHCA cases) and time from collapse to CPR initiation (missing in 40.2% of D-OHCA and 28.1% of ND-OHCA cases). This may have introduced bias, particularly in analyses involving these parameters. Additionally, the number of cases with out-of-hospital cardiac arrest attributed to drowning was quite low. This limits the robustness of the findings and prevented more detailed analysis of factors influencing outcome, like a multivariate regression analysis of D-OHCA.

Another limitation of this study is that it could not obtain data on all parameters recommended by the Utstein style for drowning (USFD) recommendations [39]. In the revised 2017 version the reporting of 22 core and 19 supplementary parameters is recommended [40]. One review found that in 14 recent papers on resuscitation in drowning, only 6–19 core (27–86%) parameters and 1–12 (5–63%) supplementary parameters were reported [7]. Another study highlighted that collecting a complete dataset according to the Utstein style is extremely time-consuming and therefore requires dedicated time and funding for data collection [41]. As the German Resuscitation Registry does not collect all parameters, this study could not report on three core parameters (precipitating event, body water type, the state of the patient at removal from the water) as well as about half of the supplemental parameters, especially not on water temperature and time of submersion, which have been shown to influence outcome [8]. Another limitation is that the method of prehospital temperature measurements is not recorded by the GRR, but may lead to significant differences [42]. On the other hand, the GRR reports on factors possibly influencing outcome such as initial rhythm or adrenaline dose that are not listed in the USFD recommendations.

## Conclusions

Drowning was a rare cause of OHCA in Germany with half of the cases occurring in summer. Although drowning patients were younger and had fewer pre-existing medical conditions, increased survival and a higher rate of favourable neurological outcome at hospital discharge compared to ND-OHCA were observed only in children up to 10 years of age. Hypothermia was associated with an unfavourable outcome in D-OHCA in the majority of cases.

## Supplementary Information

Below is the link to the electronic supplementary material.

Supplementary Material 1

## Data Availability

The datasets generated and/or analysed during the current study are not publicly available due individual data protection regulations but are available from the corresponding author on reasonable request.
