# Supplementary material for: Characteristics and outcomes of out-of-hospital cardiac arrest due to drowning – a nationwide registry-based study
Source: Scand J Trauma Resusc Emerg Med. 2025 Oct 2;33:151. doi: 10.1186/s13049-025-01469-1 (PMC12490134; doi:10.1186/s13049-025-01469-1)
Supplement: Supplementary file 1 — Supplementary Material 1 [file 13049_2025_1469_MOESM1_ESM.docx]

#### Table S1: ’Prehospital dataset’ of the German Resuscitation Registry

| **Field name** | **Expression** |
| --- | --- |
| Date of operation |  |
| Number of inhabitants in EMS |  |
| Date of birth |  |
| Age |  |
| Gender | 01=male 02=female |
| Resuscitation treatment | Has resuscitation been started? 01=Resuscitation performed 02=Death ascertained, without resuscitation  03=Resuscitation not performed, because DNR-order available 04=Resuscitation not performed, because the underlying disease is known to have no chance of recovery 05=Resuscitation not performed because futile other factors (e.g., time exceeded) present |
| Pre Emergency Status | Condition before the onset of the emergency 00 = not documented 01 = without previous illnesses  02 = previous illnesses without significant restriction of daily life 03 = previous illnesses with significant restriction of daily life 04 = normal daily life impossible 05 = patient will die within the next 24 hrs. with and without medical help |
| Suspected cause of OHCA | 01 = cardiac  02 = trauma  03 = drowning  04 = hypoxia 05 = intoxication  06 = intracerebral bleeding / subarachnoid hemorrhagie  07 = sudden infant death syndrom 08 = hemorrhage 09 = stroke 10 = metabolic 11 = other 12 =sepsis 99=not known |
| Cardiac arrest during sports activity | 01 = Yes 02 = No |
| Pre-existing conditions | 01 = cardiac 02 = lung 03 = metabolic  04 = oncological 05 = neurology  06 = Immunodeficiency |
| Collapse time |  |
| Time of emergency call |  |
| Time of alarm first responder (FR) |  |
| Time of arrival on scene FR |  |
| Time of arrival patient FR |  |
| Time of alarm ambulance |  |
| Time of EMS arrival on scene |  |
| Time of EMS arrival at the patient |  |
| Start of transport |  |
| Time of handover clinic |  |
| Time of alarm emergency physician |  |
| Time of physician arrival on scene |  |
| Time of physician arrival patient |  |
| Location of arrest | 01=home 02=nursing home 03=workplace 04=doctor's office 05=street 06=public place 07=hospital 08=mass facility 09=Other 10=educational facility 11=Sports facility, 12=birth center/facility |
| Emergency physician qualification | 01 = physician in further training 02 = specialist 03 = Specialist with additional intensive care training |
| Emergency physician Specialty | 01 = internal 02 = surgery 03 = anesthesia 04 = Pediatrics 05 = Other specialty |
| Initial ECG | 01 = bradycardia 09 = VF 10 = Pulseless electrical activity 11 = Asystole |
| Respiration at cardiac arrest | 08 = gasping 09 = apnea 10 = ventilation |
| Blood glucose |  |
| Temperature |  |
| Collapse observed | 01=lay person 02=first responder (FR) 03=EMS 04=emergency physician 05=not observed |
| Chest compressions | Start chest compressions by  01 = lay person,  02 = first responder 03 = EMS 04 = emergency physician |
| Ventilation | Start ventilation by 01 = lay person 02 = first responder 03 = EMS 04 = emergency physician |
| Defibrillator connected | Defibrillator connected by 01 = lay person 02 = first responder 03 = EMS 04 = emergency physician |
| First Defibrillation | First defibrillation performed by 01 = lay person 02 = first responder 03 = EMS 04 = emergency physician |
| Supraglottic airway device | First supraglottic airway device by. 01 = lay person 02 = first responder 03 = EMS 04 = emergency physician |
| endotracheal intubation | Endotracheal intubation by 01 = lay person 02 = first responder  03 = EMS 04 = emergency physician |
| i.v. access | First i.v. access by 01 = lay person 02 = first responder  03 = EMS 04 = emergency physician |
| First vasopressor | First vasopressor by 01 = lay person 02 = first responder  03 = EMS 04 = emergency physician |
| 1st ROSC by | First ROSC achieved by 01 = lay person 02 = first responder 03 = EMS 04 = emergency physician |
| Time of start chest compressions |  |
| Time of start ventilation |  |
| Time of connection defibrillator |  |
| Time of 1st defibrillation |  |
| Time of supraglottic airway device |  |
| Time of intubation |  |
| Time of first i.v. access |  |
| Time of first vasopressor |  |
| Time of first ROSC |  |
| Pacemaker | 05=Yes, 06=No |
| Open CPR | 01=Yes 02=No |
| central venous line | 05=Yes 06=No |
| intraosseous needle | 05=Yes 06=No |
| endobronchial medication | 01=Yes 02=No |
| Type supraglottic airway device | 03 = laryngeal mask  04 = Combitube 06 = other methods 07 = laryngeal tube 08=I-GEL |
| active cooling | active cooling in the prehospital phase 01 = Yes 02 = No |
| Cooling type |  |
| Feedback system used | 05=Yes 06=No |
| Type feedback system used |  |
| Mechanical chest compression device used | 05=Yes 06=No |
| Type mechanical chest compression device |  |
| Other technical tools | 01 = yes 02 = no |
| Type technical tool |  |
| Epinephrine |  |
| Vasopressin |  |
| Atropine |  |
| thrombolysis |  |
| sodium bicarbonate |  |
| Lipid Resuscitation |  |
| Amiodarone |  |
| Analgesic |  |
| Hypnotic / sedative |  |
| Colloid infusion |  |
| Crystalloid infusion |  |
| Hypertonic hyperoncotic infusion |  |
| Type of the first successful shock | 05 = monophasic 06 = biphasic |
| Energy of the first successful shock |  |
| Number of shocks delivered |  |
| defibrillator manufacturer |  |
| Code defibrillator |  |
| Connection of public/private accessible defibrillator | 01 = Yes 02 = No |
| Handover consciousness | 01 = analgosedated / anesthesia 02 = awake  03 = reacts to speech 04 = reacts to pain stimulus 05 = unconscious |
| Handover ECG | 01 = sinus rhythm 02 = absolute arrhythmia 03 = AV block II 04 = AV block III 05 = narrow QRS tachycardia 06 = wide QRS tachycardia 09 = ventricular flutter/fibrillation 10 = Pulseless electrical activity 11 = Asystole 12 = Pacemaker rhythm 13 = infarct ECG/STEMI |
| Handover breathing | 01 = spontaneous breathing 02 = dyspnea 03 = cyanosis 04 = spasticity 05 = Rales 06 = Stridor 07 = Airway obstruction 08 = gasping for breath 09 = apnea 10 = Ventilation 11 = hyperventilation |
| Handover GCS |  |
| Handover systolic blood pressure |  |
| Handover diastolic blood pressure |  |
| Handover heart rate |  |
| Blood glucose 2 |  |
| Handover breathing rate |  |
| Handover SpO2 |  |
| Handover exp CO2 |  |
| Handover temperature |  |
| ROSC | 01 = never ROSC 02 = ROSC |
| Hospital Admissions | 01 = No hospital admission, death at scene. 02 = hospital admission with ROSC 03 = hospital admission with ongoing resuscitation |
| Termination time of resuscitation |  |
| Complication during airway management | 06 = Airway management difficult, change of procedure 08 = Intubation difficult, more than 1 attempt 09 = coniotomy, surgical airway |
| Complications i.v. access | 03 = i.v.-access difficult, more than 2 attempts 04 = i.v.-access impossible, change of procedure necessary |
| Telephone guided CPR | 01=Yes 02=No |

CPR: cardiopulmonary resuscitation; EMS: Emergency medical service; ROSC: return of spontaneous circulation

#### Table S2: Proportion of drowning as a cause of cardiac arrest over the study period and the monthly distribution of resuscitations related to drowning.

| **Proportion of drowning as cause of OHCA over the study period** | | | | | | | | | | | | |
| --- | --- | --- | --- | --- | --- | --- | --- | --- | --- | --- | --- | --- |
| Year | 2013 | 2014 | 2015 | 2016 | 2017 | 2018 | 2019 | 2020 | 2021 | 2022 | 2023 | total |
| Percentage of drowning OHCA | 0.5% | 0.4% | 0.6% | 0.4% | 0.5% | 0.5% | 0.4% | 0.4% | 0.5% | 0.5% | 0.4% | 0.5% |
|  | | | | | | | | | | | | |
| **Seasonal distribution of D-OHCA cases** | | | | | | | | | | | | |
| Month | Jan | Feb | Mar | April | May | Jun | Jul | Aug | Sept | Oct | Nov | Dec |
| Percentage of all drowning cases | 2.5% | 4.4% | 6.3% | 5.7% | 6.0% | 17.7% | 17.4% | 14.9% | 5.7% | 6.0% | 7.0% | 6.3% |

D-OHCA: out-of-hospital cardiac arrest attributed to drowning, OHCA: out-of-hospital cardiac arrest

#### Table S3: Initial body temperature and outcome in D-OHCA according to season

| **D-OHCA** | **june-august** | **other** | **p-value** |
| --- | --- | --- | --- |
|  | **n=158** | **n=158** |  |
|  | **n (%)** | **n (%)** |  |
| *Initial temperature (°C)* |  |  |  |
| <24 | 2 (1.3) | 17 (10.8) | **<0.001** |
| 24 - <28 | 5 (3.2) | 16 (10.1) |  |
| 28 - <32 | 22 (13.9) | 17 (10.8) |  |
| 32 - 35 | 25 (15.8) | 12 (7.6) |  |
| >35 | 18 (11.4) | 12 (7.6) |  |
| missing | 86 (54.4) | 84 (53.2) |  |
| *Survival* |  |  |  |
| At hospital discharge | 27 (17.1) | 20 (12.7) | **0.268** |
| Favourable Outcome at hospital discharge | 20 (12.7) | 12 (7.6) | **0.136** |

D-OHCA: out-of-hospital cardiac arrest attributed to drowning,

#### Table S4: Multivariate regression analysis of prognostic factors for favourable outcome at hospital discharge for patients with OHCA

|  | **OR (95% CI)** | **p-value** |
| --- | --- | --- |
| *sex* |  |  |
| female* |  |  |
| male | 1.05 (0.97-1.13) | 0.209 |
| *age* |  |  |
| <60 years* |  |  |
| 60-<80 years | 0.64 (0.60-0.70) | p<0.001 |
| >= 80 years | 0.30 (0.27-0.33) | p<0.001 |
| *witnessed* |  |  |
| no* |  |  |
| lay person | 2.10 (1.92-2.29) | p<0.001 |
| EMS | 2.89 (2.55-3.28) | p<0.001 |
| *location of arrest* |  |  |
| home and other* |  |  |
| nursing home | 0.64 (0.54-0.76) | p<0.001 |
| working place | 1.74 (1.46-2.07) | p<0.001 |
| doctor’s office | 2.20 (1.81-2.66) | p<0.001 |
| public place | 1.82 (1.63-2.04) | p<0.001 |
| medical institution | 1.51 (1.19-1.92) | p<0.001 |
| mass event | 1.67 (0.88-3.19) | 0.118 |
| others | 1.46 (1.33-1.61) | p<0.001 |
| sports facility | 2.41 (1.95-2.97) | p<0.001 |
| *First documented* |  |  |
| VF/pVT* |  |  |
| pulseless electrical activity | 0.38 (0.35-0.42) | p<0.001 |
| asystole | 0.12 (0.11-0.13) | p<0.001 |
| *bystander CPR* |  |  |
| no* |  |  |
| yes | 1.50 (1.36-1.64) | p<0.001 |
| *Time between collapse and start of CPR* |  |  |
| unknown* |  |  |
| <2 min | 3.41 (3.05-3.80) | p<0.001 |
| 2-<10 min | 2.24 (2.02-2.47) | p<0.001 |
| >=10 min | 1.66 (1.46-1.89) | p<0.001 |
| *mechanical CPR* |  |  |
| no* |  |  |
| yes | 0.60 (0.53-0.68) | p<0.001 |
| *pre-hospital adrenaline dose* |  |  |
| no adrenaline application* |  |  |
| <2mg | 0.54 (0.50-0.60) | p<0.001 |
| 2-<4mg | 0.26 (0.24-0.29) | p<0.001 |
| 4-<6mg | 0.11 (0.10-0.13) | p<0.001 |
| >=6mg | 0.06 (0.05-0.07) | p<0.001 |
| *pre emergency status (PES)* |  |  |
| unknown* or severe disease |  |  |
| PES 1 | 1.82 (1.63-2.02) | p<0.001 |
| PES 2 | 1.65 (1.53-1.78) | p<0.001 |
| *Pre-hospital application*  *of amiodarone* |  |  |
| no* |  |  |
| yes | 1.54 (1.41-1.68) | p<0.001 |
| *Presumed aetiology of OHCA* |  |  |
| cardiac or unknown* |  |  |
| trauma | 0.22 (0.16-0.30) | p<0.001 |
| **drowning** | **1.07 (0.62-1.83)** | **0.811** |
| hypoxia | 1.22 (1.09-1.37) | p<0.001 |
| others non-cardiac | 0.99 (0.87-1.13) | 0.885 |

* reference category

OHCA: out-of-hospital cardiac arrest, CPR: cardiopulmonary resuscitation, EMS: Emergency Medical Service
